# Fetuin-A is a predictor of mortality in organophosphate poisoning

**DOI:** 10.1097/MD.0000000000043945

**Published:** 2025-08-15

**Authors:** Sedat Ozbay, Abuzer Coskun, Orhan Ozsoy, Gulsum Kavalci, Cemil Kavalci

**Affiliations:** a Emergency Department, Sivas Numune Hospital, Sivas, Turkey; b Emergency Department, Bagcilar Training and Research Hospital, Istanbul, Turkey; c Anaesthesia and Reanimatian, Antalya Training and Research Hospital, Antalya, Turkey; d Emergency Department, Antalya Training and Research Hospital, Antalya, Turkey.

**Keywords:** emergency, fetuin-A, organophosphate poisoning

## Abstract

Organophosphate (OP) poisoning is a serious health problem that causes morbidity and mortality in developing countries. It is important to determine biomarkers and exposures to diagnose organophosphate poisoning. Fetuin-A is a negative, acute-phase reactant. We aimed to investigate whether fetuin-A could be used to predict mortality in organophosphate poisoning. Consecutive patients aged > 18 years who were admitted to the emergency department were included in this study. Fetuin-A and pseudocholinesterase levels, hemograms, and biochemical blood parameters of patients were documented. Patients were divided into 2 groups: survivors (group 1) and those who died (group 2) due to organophosphate poisoning. Chi-square test and Mann–Whitney *U* test were used to compare the 2 groups. To determine the factors affecting mortality, the statistically significant parameters determined in the univariate analysis were included in the multivariate logistic regression risk model. Statistical significance was set at *P* < .05. The mean age of all cases was 38.05 ± 11.29 years, and 88 (40.5%) were female. The serum fetuin-A, pseudocholinesterase, and lactate levels of patients in Group I were 148.03 ± 18.93 ng/mL, 5184.58 ± 1793.41 U/L, and 2.84 ± 1.26 mmol/L, respectively. Whereas, in Group 2, these values were 101.91 ± 5.29 ng/mL, 1034.06 ± 59.66 U/L, and 6.96 ± 0.56 mmol/L, respectively (*P* < .001). However, in logistic regression analysis, fetuin-A, lipase, and lactate were identified as prognostic markers. The specificity and sensitivity of fetuin-A, lipase, and lactate levels were high in ROC curve analysis. Fetuin-A is a potential prognostic marker for organophosphate poisoning.

## 1. Introduction

Organophosphate (OP) poisoning is a major health problem that causes morbidity and mortality in the rural areas of developing countries.^[1,2]^ Approximately 3 million poisonings occur annually, resulting in 200,000 deaths.^[2,3]^ Approximately 15% of the people die.^[2]^ Organophosphates inhibit carboxylic ester hydrolases such as acetylcholinesterase and pseudocholinesterase in the body. Acetylcholine accumulates as a result of inhibition by these enzymes. The clinical signs and symptoms of OP poisoning are due to the accumulation of acetylcholine in nerve junctions. Symptoms of cholinergic crisis in the acute phase include nausea, vomiting, diarrhea, abdominal cramps, urinary incontinence, miosis, salivation, lacrimation, bronchorrhea, bradycardia, hypotension, fasciculation, muscle paralysis, dizziness, confusion, seizures, coma, and respiratory failure.^[4]^ Organophosphate compounds stored in adipose tissue that are not metabolized are remobilized and enter the circulation within 1 to 4 days. Organophosphate compounds that recirculate cause acute toxicity.^[5]^ There are many mechanisms underlying these effects; however, inflammatory and anti-inflammatory effects are important. Inflammatory responses are regulated by both pro-inflammatory and anti-inflammatory cytokines, which are related to the duration of exposure to OPs.^[6]^ After OF exposure, there is an increase in pro-inflammatory markers, but a lack of modulation of immune regulatory and anti-inflammatory markers. Microglial cells rapidly show a pro-inflammatory phenotype.^[7]^ A cohort study of 2 separate populations from Pakistan and Cameroon found that IL-6, IL1-β, and TNF-α levels were higher in chronically OP-exposed groups than in the unexposed group, and a statistically significant increase was observed. Serum esterase/lactonase and paraoxyglucoseparaoxonase 1 play an important role in the hydrolysis of various OPs. It was found to have a causal relationship with acetylcholinesterase and pro-inflammatory biomarkers [IL-6, TNF-α, and C-reactive protein] in OF-exposed individuals.^[8]^ Generally, OPs can induce an inflammatory response that promotes cell death.^[6]^

Various markers have been used to diagnose and predict exposure and outcomes in patients with OP poisoning.^[9–12]^ For this reason, pseudocholinesterase continues to be used in many places for diagnostic purposes and as a prognostic indicator of mortality.^[13]^ Therefore, this study aimed to determine whether fetuin-A could be a marker for OP by taking advantage of its pro-inflammatory and anti-inflammatory effects. Fetuin-A is an acute-phase reactant with plasma glycoprotein structure. It consists of 2 different protein chains: an A chain of 282 amino acids and a B chain of 27 amino acid residues.^[14]^ The expression of this gene has been observed in embryonic cells, adult hepatocytes, fat cells, and monocytes. Fetuin-A binds various receptors and exerts a wide range of physiological and pathological effects. In addition, it acts as a regulator of calcium metabolism, osteogenesis, the insulin signaling pathway, ectopic calcification, protease inhibition, inflammatory mediation, anti-inflammatory activity, atherogenicity, and lipid formation. The glycoprotein is negatively charged and can be found in both blood and extracellular fluid. It has a long half-life of several days.^[14,15]^ Fetuin-A has a complex role in regulating inflammatory responses, and increasing evidence suggests both pro-inflammatory and anti-inflammatory properties.^[16]^ It is well known that fetuin-A is a negative acute-phase protein that declines rapidly in response to acute inflammation. The mechanism by which serum fetuin-A levels decrease is multifactorial, including decreased hepatic production, increased excretion, or enhanced destruction in response to inflammatory cytokines.^[17]^ Inflammatory cytokines released by the toll-like receptor-mediated immune system inhibit hepatic fetuin-A synthesis.^[18]^ Therefore, fetuin-A exhibits anti-inflammatory properties and acts as a protective agent against lethal systemic inflammation.^[19]^

The treatment for OP poisoning has remained largely unchanged for over 5 decades.^[20]^ The primary focus of treating OP substances should be to ensure sufficient respiration, oxygen supply, and ventilation as well as to stabilize the functioning of the heart and lungs by counteracting excessive muscarinic effects.^[21]^ Following stabilization, oximes such as pralidoxime or obidoxime are recommended to have the ability to reactivate acetylcholine. Furthermore, some researchers have reported the use of a combination of intravenous lipid emulsion (IVLE) and charcoal hemoperfusion as a potential treatment for patients with severe OP poisoning, but this suggestion is based on individual cases.^[22–25]^

This study aimed to investigate whether fetuin-A can be used to predict mortality by utilizing its inflammatory and anti-inflammatory effects in organophosphate poisoning.

## 2. Material and methods

### 2.1. Study design and population

#### 2.1.1. Research context

This cross-sectional descriptive study included patients aged >18 years who were admitted to the emergency department of Sivas Numune Hospital because of organophosphate poisoning between 2018 and 2022. All cases of OP poisoning were associated with suicide and accidental exposure. Patient diagnoses, hospitalization dates, contact information, demographics, and clinical and laboratory data were included in the hospital automation system.

#### 2.1.2. Ethical consideration

This study was approved by the Health Sciences University Bagcilar Training and Research Hospital Non-Interventional Clinical Research Ethics Committee, Date: August 8, 2023, and Decision No 2023/08/04/039. Participants were assured that their personal, contact, and identity data would not be shared in any way. All stages of this study were conducted in accordance with the provisions of the Helsinki Declaration of Research Projects.

#### 2.1.3. Sample size

Five thousand fifty four patients were admitted to our emergency department due to drug and chemical poisoning between the dates specified in the study. After excluding non-organophosphate poisonings before the study, 217 patients were included with a 5% acceptable margin of error and a 95% confidence interval analysis in the power analysis performed with the G*power 3.1.9.4 program.

#### 2.1.4. Inclusion and exclusion criteria

Patients who applied to the emergency department with a diagnosis of OP between the specified dates were included in the study. As the patients were young, no comorbidities that would affect the results were detected. Patients who met these criteria, including electrocardiography, arterial blood gas, and patients over 18 years of age, were included in the study. Exclusion criteria included patients under 18 years of age, electrocardiography, laboratory results, registration information, pancreatitis, renal failure, heart disease, cerebrovascular disease, and a history of other substance use. We did not include patients with these characteristics. Blood samples were collected for venous blood gas, serum lactate, pseudocholinesterase, hemogram, biochemical parameters, and fetuin-A levels from OP poisoning patients admitted to the emergency department, and those whose results were recorded in the system were included in the study.

#### 2.1.5. Data collection

Diagnoses, admission dates, contact information, and demographic, clinical, and laboratory data were included in our hospital’s registration system. When these patients were admitted to the emergency department, age, sex, exposure status, venous blood gas, serum lactate, blood sugar, hemogram, and blood samples were collected for biochemical analysis. Additionally, complaints from the emergency department were recorded. Electrocardiography was performed, and rhythms were noted. The mortality status of the patients was determined. Patients were divided into 2 groups: survivors (group 1) and deaths (group 2) due to organophosphate poisoning. All cases of mortality were in-hospital deaths due to emergency admission or intensive care unit admission in the acute phase. The follow-up and readmission records of the patients for 3 months after exposure to acute OP poisoning were reviewed. The clinical and demographic characteristics of the patients were compared between the groups.

### 2.2. Laboratory design

Hemogram blood samples were measured using a Sysmex DI-60 CBC Analyzer (Istanbul, Turkey). Blood samples were analyzed using Beckman Coulter Automated AU-680 (Beckman Coulter, Inc., Fullerton). The hemogram and biochemical results were studied at 45 to 60 minutes. The lactate levels of the patients were determined by venous blood gas analysis using an Acobas® b221 Blood Gas System (Roche, Basel, Switzerland). The normal lactate range is 0.5 to 1.6 mmol/L, results greater than these values were considered significant.

For feutin-A, blood samples were centrifuged at 4000 rpm (revolutions per minute) for 5 minutes and their serum was separated. Serums were stored in Eppendorf tubes at −80°C. Serum fetuin-A levels were measured using a sandwich enzyme-linked immunosorbent assay (ELISA), according to the manufacturer’s instructions (Human Fetuin-A ELISA Kit, Cat. No. EH0218, Wuhan Fine Biological Technology, Wuhan, China). The normal range for fetuin-A was 140 to 247 ng/mL, and values above and below this range were considered statistically significant.

For serum pseudocholinesterase, samples collected in biochemical tubes were studied by spectrophotometry for approximately 2 hours (Beckman Coulter Automated AU-680, Beckman Coulter, Inc., Fullerton, CA, USA). The normal range for pseudocholinesterase was 5320 to 12,900 U/L. Values below and above the threshold were considered statistically significant.

Electrocardiography: A 12-lead ECG was obtained with a bedside Cardiofax ECG-9132K (Nihon Kohden, Tokyo, Japan) within 20 minutes of the patient’s admission to the emergency department.

### 2.3. Statistical analysis

The data collected in this study were analyzed using the SPSS software package (version 20.0; SPSS Inc., Chicago). A one-sample Kolmogorov–Smirnov test was used to examine whether the variables had a normal distribution. As the data did not have a normal distribution, the Mann–Whitney *U* test was used to analyze the differences between the groups. Chi-square analysis was used to compare the relationships between categorical variables. Binary Logistic Regression was used to perform univariate and multivariate analyses of variables. Univariate analysis was performed to evaluate the correlation between patient groups and factors. Statistically significant parameters determined in the univariate analysis were included in the multivariate logistic regression risk model to determine factors affecting mortality. Receiver operating characteristic (ROC) curve analysis was used to determine the sensitivity and specificity of fetuin-A, pseudocholinesterase, and lactate levels for predicting death. Statistical significance was determined at *P* < .05.

## 3. Results

### 3.1. Demographic and clinical characteristics

All cases included in the study had a mean age of 38.05 ± 11.29 years; 88 (40.5%) of the cases were female, and the age distribution was 20 to 64 years (*P* = .554). The demographic and clinical characteristics of the patients are summarized in Table 1.

**Table 1:** Demographic and clinical characteristics of patients.

Baseline characteristics	All patients, n:217, mean ± SD
Age, years	38.05 ± 11.29
Gender	
Female/male (%)	88 (40.5)/129 (59.5)
Symptoms/signs n (%)	
Salivation	135 (62.2)
Lacrimation	131 (60.4)
Urination	91 (41.9)
Defecation	68 (31.3)
Nausea	217 (100)
Vomiting	202 (93.1)
Miosis	118 (54.4)
Sweating	176 (81.1)
Bronchospasm	46 (21.2)
Bronchorrhea	23 (10.6)

SD = standard derivation.

### 3.2. Laboratory and electrocardiographic analyses

Laboratory results were remarkable in 33 (15.21%) mortality cases (Table 2). The average age of these patients was 46.18 ± 11.26 years, 13 (39.4%) were female (*P* ≤ .001), and no relationship between sex and mortality was detected (*P* = .883). All patients died in the hospital (*P* = .028). Compared to the survivors of this group, the glucose level was 202.61 ± 19.62 mg/dL (*P* < .001), pseudocholinesterase 1034.06 ± 59.66 U/L (*P* < .001), fetuin-A 101.91 ± 5.29 ng/mL (*P* < .001), amylase 187.73 ± 44.29. U/L (*P* < .001), lipase was 336.91 ± 60.24 U/L (*P* < .001) and lactate was 6.96 ± 0.56 mmol/L (*P* < .001). Mortality was observed in 19 (12.7%) patients who were accidentally exposed to OP poisoning and 14 (20.9%) patients who attempted suicide. It was determined that 27 (81.8%) patients with mortality had bradycardia (*P* < .001). Additionally, mortality was more frequent in patients who received atropine and pralidoxime for the second time (*P* < .001; Table 2).

**Table 2:** Basic characteristics and laboratory results according to groups.

Variables		Group 1, n:184, mean ± SD	Group 2, n:33, mean ± SD	*P*-value
Demographic findings	Age, years	36.59 ± 10.69	46.18 ± 11.26	<.001*
	Gender, female/male	75/109	13/20	.883**
Laboratory results	Glucose, mg/dL	142.98 ± 37.72	202.61 ± 19.62	<.001*
	Amylase, U/L	125.69 ± 38.75	187.73 ± 44.29	<.001*
	Lipase, U/L	212.48 ± 52.52	336.91 ± 60.24	<.001*
	Lactate, mmol/L	2.84 ± 1.26	6.96 ± 0.56	<.001*
	Pseudocholinesterase, U/L	5184.58 ± 1793.41	1034.06 ± 59.66	<.001*
	Fetuin-A, ng/mL	148.03 ± 18.93	101.91 ± 5.29	<.001*
Exposure	Accident	131 (60.4)	19 (8.8)	.151
	Suicide	53 (24.4)	14 (6.5)	
Electrocardiography	Normal sinus rhythm	110 (50.7)	1 (0.5)	<.001**
	Sinus tachycardia	25 (11.5)	3 (1.4)	
	Sinus bradycardia	43 (19.8)	27 (12.4)	
	Atrial fibrillation	6 (2.8)	2 (0.9)	
Treatment	AC + A + PAM	148 (80.4)	13 (39.4)	.001**
	Re-A + PAM	16 (8.7)	14 (42.4)	
	Plasmapheresis	14 (7.6)	5 (15.2)	
	Lipid	6 (3.3)	1 (3)	

A = atropine, AC = activated carbon, mg/dL = milligram/deciliter, mmol/L = millimoles/liter, ng/mL = nanogram/milliliter, PAM = pralidoxime, pg/mL = picogram/milliliter, SD = standard deviation, U7L = unit/liter.

*P* < .05 indicates statistical significance.

* Mann–Whitney *U* test was used for other comparisons.

** Chi-square test was used for gender, exposure, electrocardiography and treatment.

### 3.3. Logistic regression univariate and multivariate analyses

While age and lactate levels were found to be insignificant in the univariate logistic analysis, glucose, pseudocholinesterase, fetuin-A, amylase, and lipase levels were found to have a statistically significant relationship with OP poisoning. However, in the multivariate logistic regression analysis, the glucose level was found to be a prognostic indicator of OP poisoning. Age and serum glucose, pseudocholinesterase, fetuin-A, lactate, amylase, and lipase levels were found to be significant in terms of mortality in the univariate logistic analysis. However, multivariate logistic regression analysis showed that fetuin-A, lipase, and lactate levels could be prognostic parameters (Table 3).

**Table 3:** Logistic regression analysis of organophosphate poisoning and mortality with variables.

Mortality	Univariate			Multivariate		
	OR	95% Cl	*P*	OR	95% Cl	*P*
Age, years	1.080	1.042–1.119	<.001			
Blood sugar, mg/dL	1.041	1.027–1.055	<.001			
Pseudocholinesterase, U/L	0.979	0.963–0.995	.012			
Fetuin-A, ng/mL	0.796	0.714–0.997	<.001	0.717	0.709–0.748	.011
Amylase, U/L	1.033	1.022–1.043	<.001			
Lipase, U/L	1.033	1.023–1.043	<.001	1.021	0.982–1.037	.001
Lactate, mmol/L	4.146	2.601–6.607	<.001	3.893	2.487–5.441	<.001

CI = confidence interval, mg/dL = milligram/deciliter, mmol/L = millimoles/liter, ng/mL = nanogram/milliliter, OR = odds ratio, pg/mL = picogram/milliliter, SD = standard deviation, U/L = unit/liter.

*P* < .05 indicates statistical significance.

### 3.4. Receiver operating characteristic curve

**Figure 1.**
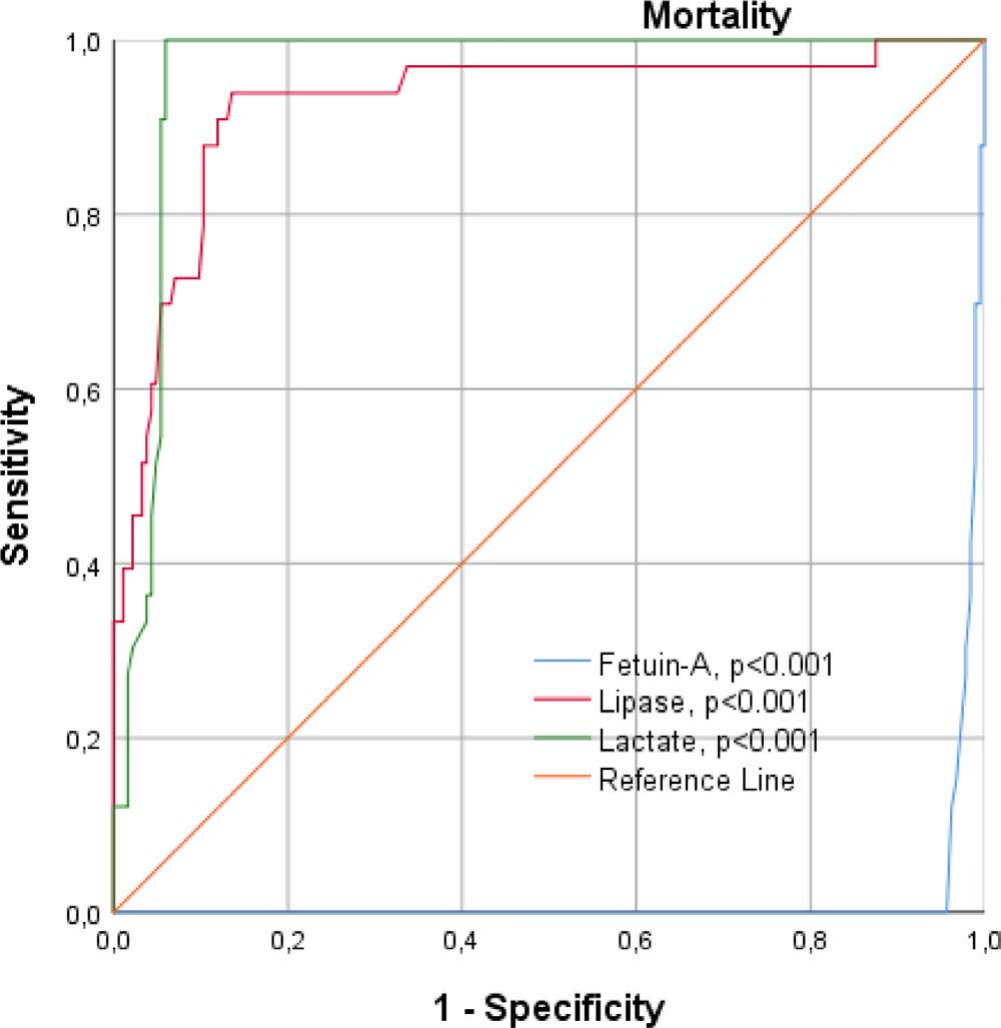
Receiver operating characteristic curve analysis; Fetuin-A, lipase, and lactate levels in mortality.

Mortality and ROC curve analyses of fetuin-A, lipase, and lactate levels are presented in Table 4 and Figure 1. It was observed that as fetuin-A serum levels decreased, prognosis worsened, and mortality increased. In the ROC curve analysis, fetuin-A sensitivity and specificity were 97.6% and 96.2%, respectively (AUC, 0.016; 95% CI: 0.002–9.032). Similarly, lipase sensitivity was 98.4% and specificity was 96.7% (AUC: 0.926; 95% CI: 0.870–0.982) (*P* < .001). As lactate levels increase, both prognosis and mortality increase. Lactate sensitivity was 97.2% and specificity was 95.9% (AUC, 0.961; 95% CI: 0.937–9.986) (*P* < .001). Changes in the 3 variables are shown in the graph.

**Table 4:** Receiver operating characteristic curve analysis.

ROC curve					
Mortality	Sensitivity (%)	Specificity (%)	AUC	95% CI	*P*-value
Fetuin-A	97.6	96.2	0.016	0.002–0.031	<.001
Lipase	96.7	94.3	0.926	0.870–0.982	<.001
Lactate	97.2	95.9	0.961	0.937–0.986	<.001

AUC = area under the curve, CI = confidence interval, ROC = receiver operating characteristic.

*P* < .05 indicates statistical significance.

## 4. Discussion

To our knowledge, for the first time in the literature, we have shown that low serum fetuin-A and high lactate levels in blood samples taken during emergency room admission with organophosphate poisoning can independently predict mortality in treated patients. According to the results of our study, low serum fetuin-A and high lactate levels are independent predictors of mortality in organic phosphate poisoning.

In all mortality cases, serum lactate and fetuin-A levels were much lower than the typical cutoff values. In addition, significant increases were observed in the serum glucose, lipase, amylase, and lactate levels. The sensitivity and specificity percentages of fetuin-A, pseudocholinesterase, and lactate were found to be high in terms of mortality. Logistic regression analysis revealed that fetuin-A, lactate, and lipase levels are the most important predictors of mortality. We also believe that fetuin-A may have predictive importance in terms of diagnosis, prognosis, and death.

The male/female ratio in our cases was 1.47/1, the average age was 38 years, and 54.4% of the patients were male. In many agricultural countries, men are at a higher risk of exposure than women are because they are more likely to be involved in agricultural activities that contain OP compounds. In the literature, the author reported that the male/female ratio was between 0.61% and 2.3%, and the average age was 32 years.^[12,26]^ The male-female ratio varies according to the socioeconomic and cultural characteristics of the country. While accidental poisoning has come to the forefront in some studies, suicide has come to the forefront in others. It was determined that 150 (69.1%) patients were accidentally exposed to OP poisoning and 67 (38.9%) were exposed to OP poisoning for suicidal purposes. In a study of 207 cases, Razwiedani et al^[26]^ reported 47.3% accidents, 50.2% suicides, and 2.4% illegal suicides. Colak et al^[27]^ reported in their study of 67 cases of suicide (67.2% suicide, 11.9% occupational exposure, and 20.9% accidental poisoning). We believe that the reason we differ from similar studies is that adolescents are vulnerable to suicide attempts, professional failures, unsuccessful relationships, conflicts with their parents, poverty, and financial difficulties in middle-aged groups.

Bradycardia, age, glucose level, lactate dehydrogenase level, and acidosis are independent predictors of mortality in patients with organophosphate poisoning.^[4,27]^ In our study, fetuin- A, lipase, and lactate levels were found to be independent predictors of mortality. This difference may be attributed to the demographic, socio-cultural, and geographical characteristics of the patients included in the study. Some researchers have reported a correlation between serum amylase, lipase, and creatinine kinase levels and severity of organophosphate poisoning.^[26,28]^ In our study, serum amylase, lipase, and lactate levels were high and acetylcholinesterase and fetuin-A levels were low in group 2 patients. As the severity of the disease increases, amylase and lipase cannot be destroyed and are detected in higher amounts in the serum, owing to the increased inhibition of cholinesterase, α-chymotrypsin, trypsin, and elastase activities. OP poisoning is associated with several abnormalities in the laboratory. Among these, hyperamylasemia, which is most frequently observed in OP poisoning cases, may be due to excessive cholinergic stimulation of the pancreas by OP compounds in acute pancreatitis.^[29]^ In a prospective study by Singh et al,^[30]^ amylase increased by 48.95%, and serum amylase showed persistent elevation during serial measurements. An important effect of OP is the development of acute pancreatitis. The incidence of acute pancreatitis in adults with OP poisoning was approximately 12%. In a study by Dungdung et al,^[10]^ lipase levels were high in 56 patients, 22 of whom had normal amylase levels, and all patients with severe OP had high lipase levels. The diagnostic accuracy of the biochemical parameters showed that serum amylase had the highest diagnostic accuracy compared to serum lipase. Lactate is an important marker for determining the level of tissue hypoxia. The normal range of blood lactate levels is 0.5 to 1.8 mmol/L. Studies have shown that patients with blood lactate levels below 2 mmol/L have low mortality and morbidity, whereas patients with blood lactate levels approaching 4 mmol/L have increased mortality and morbidity rates.^[31,32]^ Although lactate and pH values do not change the patient’s decisions regarding intensive care, hospitalization, or discharge, they have an important role in the organization of the patient’s treatment while in the emergency department.^[33]^ Patients with elevated lactate levels may be at a higher risk of significant morbidity and mortality and need a systematic approach for both diagnosis and treatment.^[34]^

Cholinergic and nicotinic symptoms were detected in 182 of 217 patients (83.9%) admitted to the emergency department with organophosphate poisoning. Among these symptoms, bronchorrhea and nausea were observed in 23 (10.6%) and 100% of cases, respectively. Gabalive et al^[28]^ reported that the rate of acute symptoms and signs ranges from 10% to 92%. Among these findings, muscle weakness was detected at a rate of 10%, and sweating at a maximum rate of 92%. In the study by Shah et al,^[35]^ these rates were bradycardia, 20% for nausea and vomiting, with 100%. Organophosphate poisoning has devastating effects; therefore, its early identification and treatment are critical. Mortality rates vary according to the amount of food intake, the method of intake, and therapy. Mortality was observed in 33 (15.2%) patients. Mortality was the result of 19 (12.7%) accidental poisonings and 14 (20.9%) suicides. The mortality rate of organophosphate poisoning ranges from 10% to 40%.^[34–36]^ Sanjay et al^[37]^ observed a link between low pseudocholinesterase levels and clinical severity. Tang et al^[38]^ found that values between 870 and 1200 on day 1 were associated with longer ventilation and increased mortality. Our results support this finding.

### 4.1. Limitations

The study’s most significant limitation was that it was a retrospective single-center study. It is challenging to ensure that all demographic, laboratory, and clinical data are correctly determined. Furthermore, the sample size was larger than that used in many other studies; however, it could have been small. In addition, a clear evaluation may not have been possible because the drugs and blood parameters used in the cases preceding poisoning were unknown. Because pseudocholinesterase and fetuin-A values were not available for all cases, the sample size may not have been optimal. Multicenter studies with larger sample sizes would yield more conclusive results.

## 5. Conclusion

Therefore, fetuin-A, lipase, and lactate levels may be useful prognostic indicators of OP poisoning. Good supportive treatment and observation can help prevent acute and delayed complications, such as monoplegia, paraplegia, and mortality.

## Author contributions

**Conceptualization:** Sedat Ozbay, Abuzer Coskun.

**Data curation:** Sedat Ozbay, Abuzer Coskun.

**Formal analysis:** Abuzer Coskun, Cemil Kavalci.

**Funding acquisition:** Sedat Ozbay.

**Investigation:** Sedat Ozbay, Abuzer Coskun.

**Methodology:** Abuzer Coskun, Gulsum Kavalci.

**Resources:** Abuzer Coskun, Orhan Ozsoy.

**Software:** Orhan Ozsoy.

**Supervision:** Sedat Ozbay, Gulsum Kavalci, Cemil Kavalci.

**Writing – original draft:** Sedat Ozbay, Abuzer Coskun.

**Writing – review & editing:** Gulsum Kavalci, Cemil Kavalci.
